# Far and Near

**Published:** 1896-10

**Authors:** 


### FAR AND NEAR.

State veterinary organizations should be started at once in Kentucky and Tennessee.

One of our readers suggests that these dull times should be fruitful periods for many of our self-made men, who yearn for a broader education, to enter our schools and take collegiate courses of instruction.

Indiana, Illinois, and Ohio should take fresh courage and strengthen their organizations for more aggressive work during the year we are now entering upon.

The report of the State Board of Agriculture for Rhode Island for 1895 contains a complete report of the papers and proceedings of the New England Cattle Commissions. We note that the experiment station and college still lack a veterinarian on the staff of the corps of instructors.

The largest single shipment of cattle ever made from the United States occurred early in September; it consisted of 2799 head. Ten steamers were necessary to carry them to London, Liverpool, and Glasgow, their destinations.

The raising of foxes in the Alaskan isles for the value of their pelts is a growing and profitable industry of our extreme Northwest.

The statement of the non-existence of tuberculosis among the cattle of the Isle of Jersey was not based upon tuberculin-tests, but by a thorough examination in 1892, by a layman, and therefore must be considered on its merits.

A half-interest in the Poland China boar, "Chief I Know," has recently been sold for $1000.

Veterinarian Bailey, of Maine, when he studies John R. Gentry's great feat, three-quarters of a mile distance at a two-minute gait, must feel a narrow escape in his prediction that there will never be in his time a two-minute racer. Gentry is but seven years old, and will, it is rumored, be shortly retired to the stud for breeding purposes only.

The once famous New England campaigner, Ezra L., 2.21¼, that recently died at Bangor, Me., at the ripe old age of twenty-three years, was buried by his owner in a velvet-covered, silver-trimmed coffin.

The Pasteur Anthrax Vaccine Company of Chicago has changed its name to the Pasteur Vaccine Company, and has recently removed to more commodious quarters, occupying some five rooms at 56 Fifth Avenue, Chicago, Ill., to which address all communications, to insure prompt attention, should be sent.

One of Pittsburg's veterinary surgeons, in addition to dentistry, claims to be a "*Foot Veterinary*," to cure pole-evil, quitter, etc. Of course, he guarantees to cure, or no pay. Experience in Europe and America is an added claim for patronage.

